# Phenotypic and clinical characterization of low density neutrophils in patients with advanced lung adenocarcinoma

**DOI:** 10.18632/oncotarget.18771

**Published:** 2017-06-28

**Authors:** Yangyang Liu, Yue Hu, Feifei Gu, Jinyan Liang, Yulan Zeng, Xiaohua Hong, Kai Zhang, Li Liu

**Affiliations:** ^1^ Cancer Center, Union Hospital, Tongji Medical College, Huazhong University of Science and Technology, Wuhan 430022, China

**Keywords:** low density neutrophils, lung cancer, EGFR, immune status

## Abstract

**Purpose:**

An immunosuppressive subgroup of neutrophils, low density neutrophils (LDNs) was reported to be closely related to several diseases. This study was designed to explore the association between LDNs and advanced lung adenocarcinoma, as well as potential mechanisms.

**Results:**

The expression levels of surface CD molecules on LDNs were different from high density neutrophils (HDNs), consistent with previous studies. The ratio of LDNs/HDNs, rather than the percentage of LDNs in peripheral blood mononuclear cells (PBMCs), was significantly higher in lung adenocarcinoma patients than healthy controls. It was also observed that the ratio decreased when patients received anti-cancer treatments, and increased when disease relapsed. Patients harboring positive epidermal growth factor receptor (EGFR) mutation had significantly higher ratios. Both the ratio and the percentage showed positive correlation with CD8+ T cells. Although significantly increased TGF-β was detected in lung adenocarcinoma patients, relationship between TGF-β and LDNs was not obvious.

**Materials and Methods:**

LDNs and HDNs levels of peripheral blood from 52 lung adenocarcinoma patients and 13 healthy controls were determined by flow cytometry. Lymphocytes and cytokines were also detected.

**Conclusions:**

Two kinds of neutrophils with different phenotypes were identified in lung adenocarcinoma patients. Besides, we found the existence of high ratio of LDNs/HDNs in these patients, which is related to disease prognosis, EGFR mutation and bad immune status.

## INTRODUCTION

Lung cancer is the leading cause of cancer-related death worldwide, with 5-year overall survival rate of 18% in the United States; adenocarcinoma makes significant contribution [1]. So it is urgent to explore new effective treatment strategies. Immunotherapy is a promising option and has showed attractive benefits in hematological neoplasms [2]. But in solid tumors, tumor microenvironment (TME) serving as a big obstacle attenuates its clinical effects. Mainly formed by extracellular matrix and stromal cells, TME contains a wealth of immunosuppressive cells such as myeloid-derived suppressor cells (MDSCs), tumor-associated macrophages, and regulatory T cells which can protect tumor from attacks [3]. Besides, growing numbers of studies indicated that neutrophils also contribute to poor prognosis [4, 5].

Neutrophils, accounting for 50%–70% of peripheral leukocytes, definitely have an immune defensive function [6, 7]. Recently, however, a subset of neutrophils was found to play an immunosuppressive role in several kinds of diseases, contradicting the traditional view. Among these studies, neutrophils are divided into high-density neutrophils (HDNs) and low-density neutrophils (LDNs) based on density variance [8–10]. Morphologically, HDNs are homogenous mature cells, but LDNs are mixed [8]. Hence, considering the coexistence of immature and mature neutrophils, LDNs are not just equal to granulocytic MDSC (G-MDSCs) for the latter being immature [8, 11]. Phenotypically, LDNs express the same CD molecular as HDNs, but CD15, CD11b and CD66b expression are higher in LDNs [10, 12]. And functionally, LDNs are immunosuppressive. IL-10 and arginase were indicated to participate, but the exact mechanism has not been clearly elucidated [13, 14]. What’s more, impaired activity of LDNs was also revealed to cause cancer permission, contrasted to conventional normal HDNs [8].

The knowledge relating to LDNs in lung cancer is insufficient. The present study was to determine whether LDNs or ratio of LDNs/HDNs are elevated in lung adenocarcinoma patients, and their correlation to clinical characteristic and immune status. Also, we tried seeking potential upstream mechanism of increased LDNs or ratio.

## RESULTS

### Subject characteristics

Three groups of patients, 21 treatment naïve, 12 recurrent unreceiving anti-cancer therapies for at least 6 months, and 19 in the interval of treatment cycles, were enrolled in our study. The control group was composed of another 13 healthy individuals. Baseline characteristics of all subjects were presented in Table 1. There was no significant difference as for age and sex distributions among the four groups (*p* = 0.848, *p* = 0.056 respectively). Disease stage was evaluated according to the Union for International Cancer Control (UICC) tumor node metastasis (TNM) staging system. Most of the patients (88.5%) were stage IV, and only 6 individuals (11.5%) stage III. The information on epidermal growth factor receptor (EGFR) mutation was available for 36 subjects, 26 mutation positive and 10 negative. In treated group, 8 patients received chemotherapy, 4 radiotherapy, 4 targeted therapy and 3 concurrent chemoradiotherapy.

**Table 1:** Basic characteristics of subjects (*n* = 65)

Characteristic	Patients^a^			
	Untreated	Recurrent	Treated	Control
Subjects number	21	12	19	13
Median age, years (range)	60 (32–74)	58.5 (53–77)	61 (39–75)	58 (49–75)
Gender				
Male	8	6	14	10
Female	13	6	5	3
Tumor stage				
III	2	2	2	/
IV	19	10	17	/
EGFR^b^ status				
Mutation positive				/
Exon 19	6	3	4	
Exon 21	2	6	5	
Mutation negative	4	0	6	/
Unknown	9	3	3	/

^a^Untreated patients did not receive any therapies, recurrent patients had stopped anti-cancer treatment for at least 6 months, and treated ones were under treatment. ^b^EGFR, epidermal growth factor receptor

### Phenotype of HDNs and LDNs

**Figure 1:**
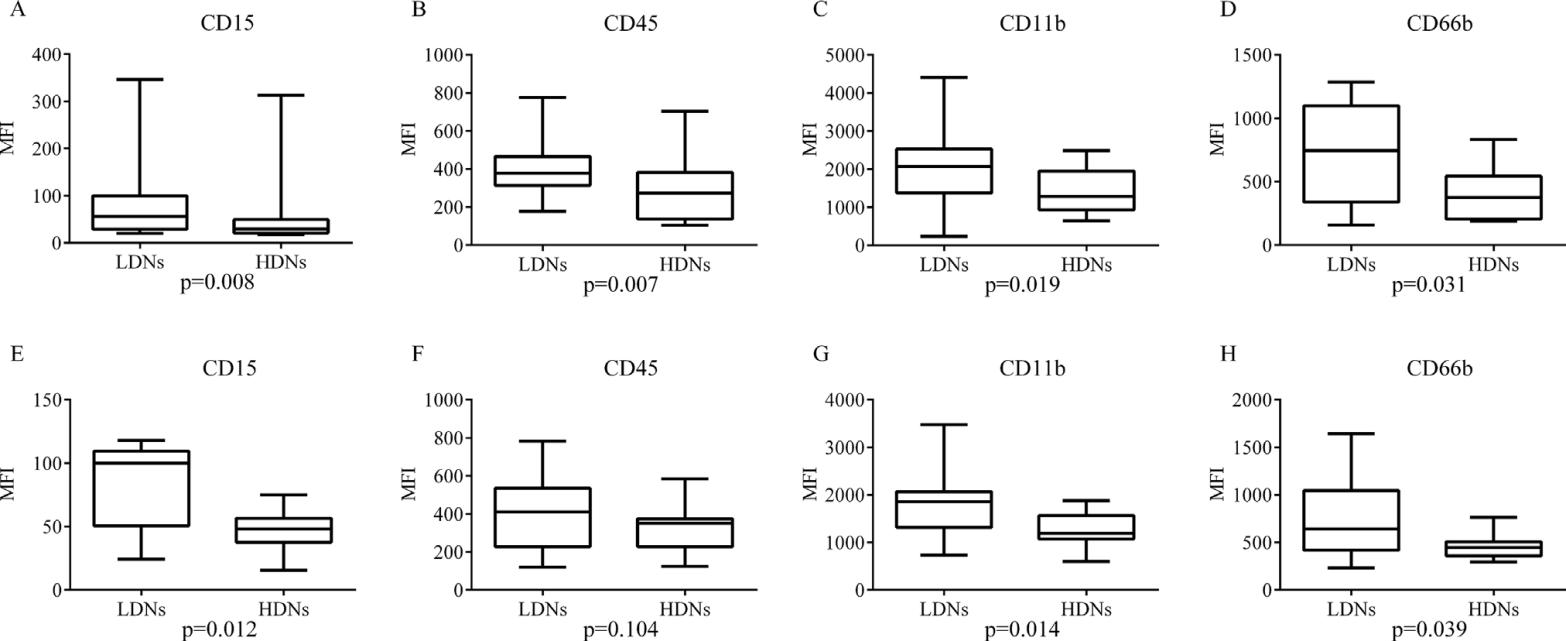
Expression levels of four surface CD molecules in LDNs and HDNs LDNs and HDNs from untreated patients (**A**–**D**) and healthy controls (**E**–**H**) were detected by flow cytometry for expression level of CD15, CD45, CD11b and CD66b. Statistical significance was determined by a two-tailed Mann-Whitney test. Box = interquartile range and median; whiskers = range.

According to density diversity, we divided granulocytes in two groups, HDNs coexisted with granulocyte-erythrocyte fractions and LDNs concomitant with PBMCs. Both LDNs and HDNs express neutrophil marker. Using flow cytometry, SSC and CD45 were combined to roughly selected granulocytes, then CD15 was applied to identify HDNs and LDNs in granulocyte-erythrocyte fractions and PBMCs respectively. Previous studies also indicated that these cells differ phenotypically in HIV, SLE and asthma patients [10, 12, 15]. So in this study, we compared expression levels of CD15, CD45, CD11b and CD66b in the two group cells. For untreated patients, the mean fluorescence intensities (MFI) of CD15, CD45, CD11b and CD66b were all significantly greater on LDNs than HDNs (Figure 1A–1D). Results from the relapsing and treated groups were similar (data not shown). While in healthy controls, the LDNs only expressed higher levels of CD15, CD11b and CD66b, and there was no significant difference in the MFI in CD45 (Figure 1E–1H).

### LDNs frequency and ratios of LDNs/HDNs

**Figure 2:**
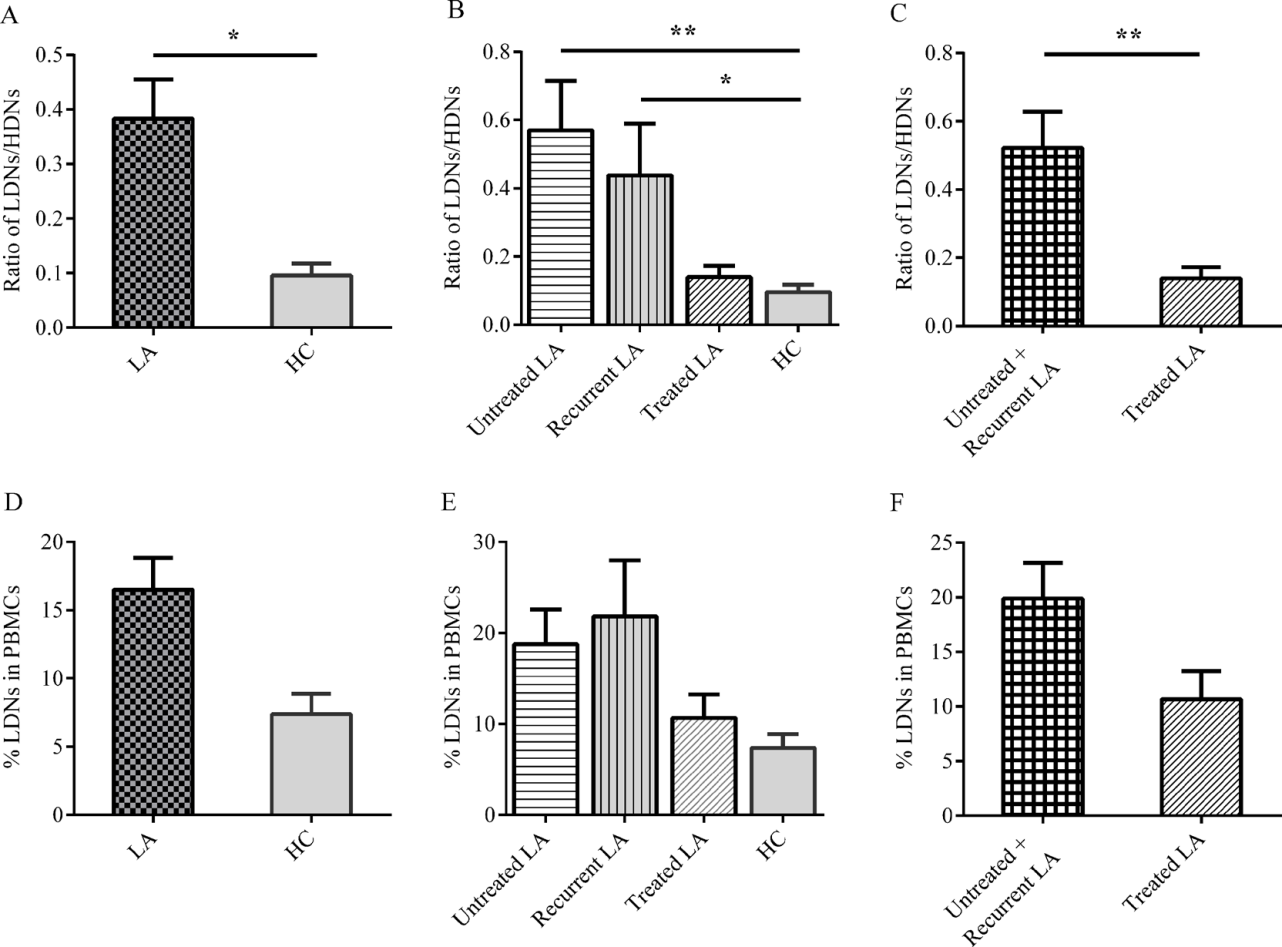
Percentage of LDNs in PBMCs and ratio of LDNs/HDNs analyzed in lung adenocarcinoma (LA) patients and healthy controls (HC) The ratio of LDNs/HDNs in total LA patients, treatment subgroup and HC (**A**–**C**). Treated means patients received anti-cancer therapies recently. LDNs percentage in PBMCs was also analyzed in LA patients (**D**–**F**). Statistical significance was determined by a two-tailed Kruskal-Wallis test (B, E), others were by a two-tailed Mann-Whitney test. *P < 0.05, **P < 0.01.

There are 52 adenocarcinoma subjects. Unlike other studies, there were no significantly more LDNs in PBMCs comparing patients to healthy controls, and therapy history did not impact the LDNs frequency, either (Figure 2D–2F). Following recent horizon that the immune status could be reflected better in the competition of positive and negative cells [16], we measured the ratios of LDNs/HDNs. Adenocarcinoma individuals possessed significantly increased ratios to normal people (0.383 ± 0.073 vs. 0.080 ± 0.022, *P* = 0.015) (Figure 2A). When we took intervention history into consideration, the ratios were significantly higher in untreated (0.571 ± 0.144) or recurrent patients (0.438 ± 0.151) as compared to controls (0.080 ± 0.022, *P* = 0.005, Figure 2B), and the ratios in patients undergoing treatment were significantly decreased as compared to other patients (0.139 ± 0.034, *P* = 0.005, Figure 2C). To exclude the inconsistence of peripheral blood and tumor microenvironment, we analyzed LDNs in malignant pleural effusion (MPE). The ratios of LDNs/HDNs, not LDNs frequency, in lung adenocarcinoma patients MPE and blood were comparably significantly higher than healthy controls blood (Supplementary Figure 1).

Difference was not significant between stage III and IV patients for LDNs frequency and LDNs/HDNs ratio (*P* = 0.860, *P* = 0.856, respectively). In treated group, different therapy methods also did not generate significant variance between each other (*P* = 0.990, *P* = 0.802, respectively)

### EGFR mutation

**Figure 3:**
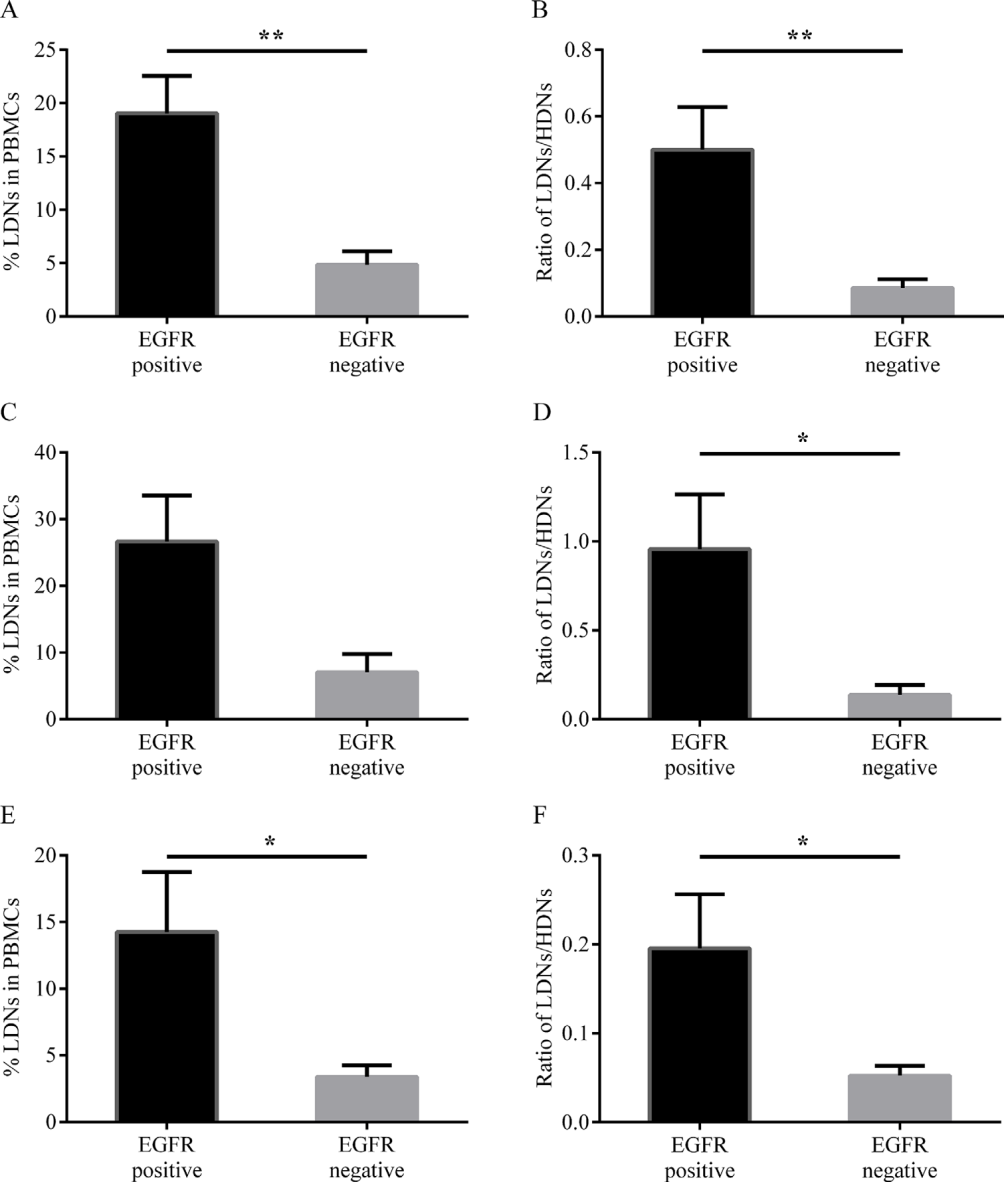
Percentage of LDNs in PBMCs (**A**, **C** and **E**) and ratio of LDNs/HDNs (**B**, **D** and **F**) from patients (*n* = 36) with different EGFR status. Total patients (A, B), treatment naïve patients (C, D) and treated patients (E, F) with EGFR mutation data. Statistical significance was determined by a two-tailed Mann-Whitney test. *P < 0.05, **P < 0.01.

A new research revealed that intra-tumoral neutrophils are related to EGFR mutation in lung cancer [17], so whether similar clinical association existed in our study? We merged untreated and recurrent patients together for their close characteristics of LDNs (Figure 2B). 36 patients with available mutation information were distributed uniformly between each groups (Table 2, *P* = 0.260), therefore we performed an integrally analysis according to EGFR gene status. As shown in Figure 3A, 3B, both LDNs frequency and ratio of LDNs/HDNs in EGFR mutation positive subjects were significantly higher than negative ones (*P* = 0.005, *P* = 0.004, respectively). Subgroup analysis showed that although LDNs frequency was significantly higher in EGFR mutation positive patients in treated group (*P* = 0.018, Figure 3E), the difference was not obvious in treatment naïve group (*P* = 0.105, Figure 3C). In both subgroups, LDNs/HDNs ratios were significantly higher in mutation positive patients (*P* = 0.049, *P* = 0.036, respectively, Figure 3D, 3F). Difference between patients harboring gene mutation in exon 19 and 21 of EGFR was not obvious (data not shown).

**Table 2:** Distribution of EGFR status in patients

Treatment history	EGFR status	
	Positive	Negative
Untreated + recurrent	17	4
Under treatment	9	6

The distribution is statistically uniform (*P* = 0.114), determined by Fisher’s exact test.

### Correlation between neutrophils and lymphocytes

**Figure 4:**
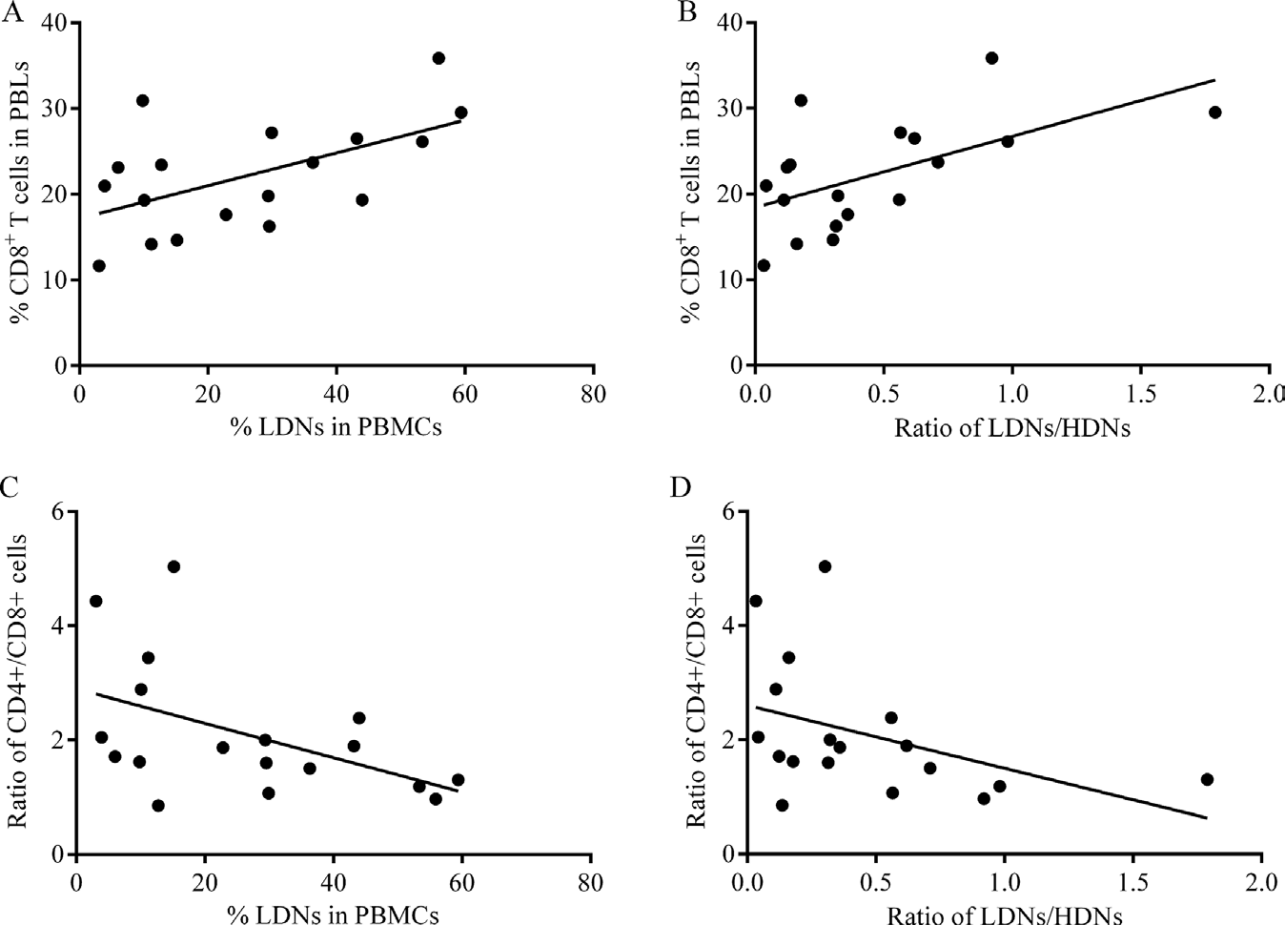
Correlation between CD8^+^ T cells percentage and LDNs frequency (**A**, **C**) or LDNs/HDNs ratio (**B**, **D**) in patients (*n* = 18). Statistical significance was determined by a Spearman’s rank test.

Lymphocytes could more immediately reflect immune status. To assess the correlation between lymphocytes and LDNs, we concurrently detected peripheral blood lymphocyte (PBLs) subtypes in 18 patients. The percentages of CD4^+^ T cells and NK cells were not found to statistically correlate with frequency of LDNs or LDNs/HDNs ratio (Table 3). While both frequency of LDNs and LDNs/HDNs showed positive correlations with frequency of CD8^+^ T cells, and inverse correlations with ratio of CD4^+^/CD8^+^ T cells (Figure 4, Table 3).

**Table 3:** Correlation between neutrophils and lymphocytes

Correlation	*r*	95% CI	*P*	Significant
LDNs vs. CD4^+^ T	−0.404	−0.739 to 0.093	0.097	No
LDNs vs. CD8^+^ T	0.503	0.032 to 0.791	0.034	Yes
LDNs vs. NK^a^	−0.143	−0.360 to 0.582	0.570	No
LDNs vs. CD4^+^/CD8^+^ T	−0.511	−0.795 to −0.043	0.030	Yes
LDNs/HDNs vs. CD4^+^ T	−0.404	−0.739 to 0.093	0.097	No
LDNs/HDNs vs. CD8^+^ T	0.589	0.154 to 0.833	0.010	Yes
LDNs/HDNs vs. NK	0.170	−0.336 to 0.600	0.499	No
LDNs/HDNs vs. CD4^+^/CD8^+^ T	−0.548	−0.813 to −0.094	0.019	Yes

The correlation between LDNs frequency or LDNs/HDNs ratio with lymphocytes subgroups frequency was analyzed (*n* = 18). Statistical significance was determined by a two-tailed Spearman’s rank test. ^a^NK, natural killer cells.

### Cytokine in plasma of lung cancer patients

**Figure 5:**
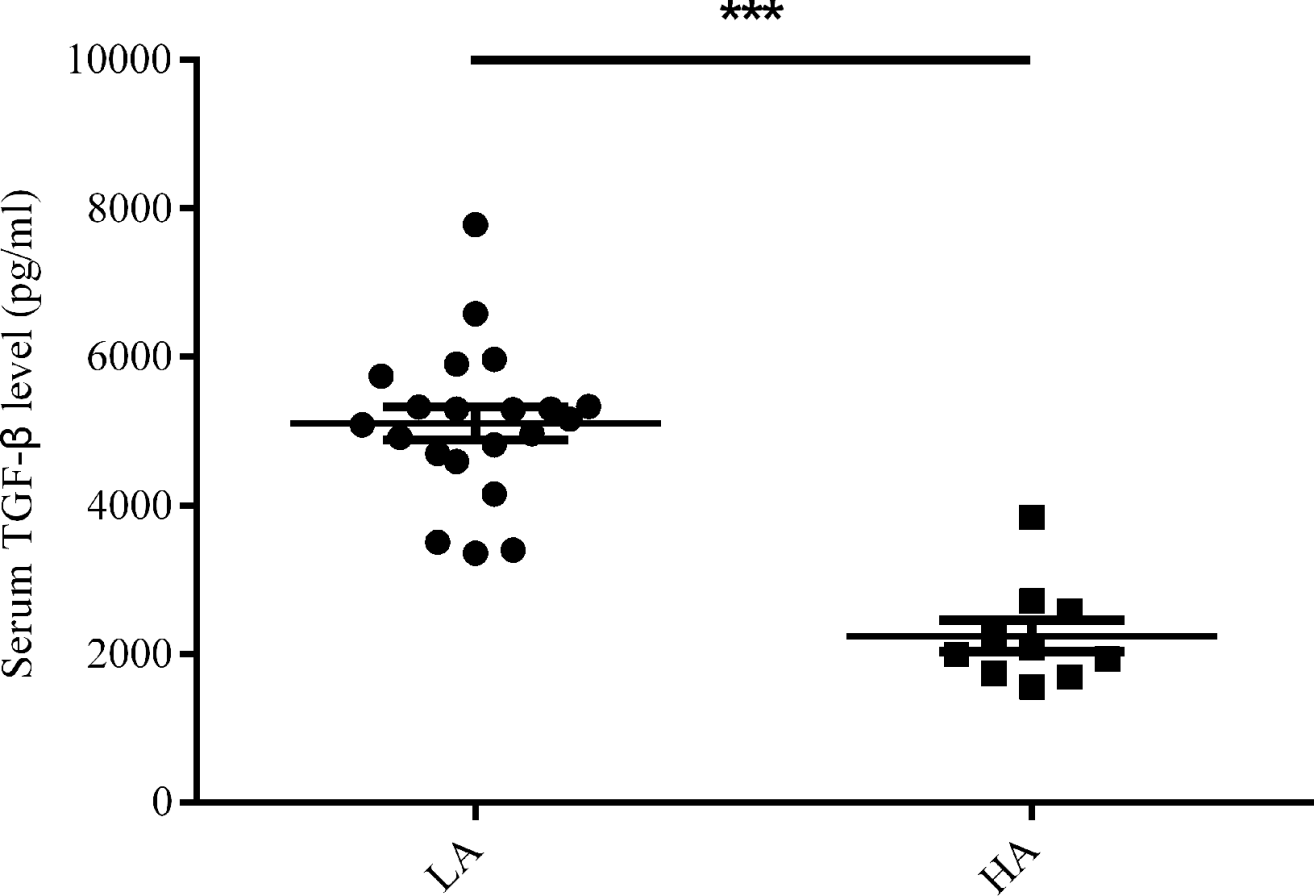
Serum TGF-β level (pg/ml) in untreated lung adenocarcinoma (LA) patients (*n* = 21) and healthy controls (HC) group (*n* = 10) Statistical significance was determined by a two-tailed Mann-Whitney test. ***P < 0.001.

Two cytokines, IL-17 and TGF-β were proved to be responsible to increased LDNs [8, 18]. So we detected their content in plasma of treatment naïve lung adenocarcinoma patients. As a result, IL-17 seldom existed in all subjects, let alone statistical difference (data not shown). For TGF-β, the level in plasma of patients was significantly higher than healthy controls (5.102 ± 0.226 × 10^3^ vs. 2.239 ± 0.213 × 10^3^ pg/ml, *P* < 0.001) (Figure 5). Nevertheless, neither LDNs frequency nor the ratio of LDNs/HDNs showed correlation with TGF-β content (*P* = 0.614, *P* = 0.610, respectively).

## DISCUSSION

A growing body of evidence suggested that an immunosuppressive subset of neutrophils, LDNs, are associated with various diseases, including HIV, SLE, tuberculosis and cancer [8–10, 12, 15, 19]. This study, for the first time, researched LDNs in lung adenocarcinoma patients. It is commonly believed that immune balance is of key importance for tumor development, therapy and prognosis [3, 16, 20]. As pro-tumor LDNs and anti-tumor HDNs, the ratio of them represents part of the balance. Consistent with this view, here we demonstrated it was the LDNs/HDNs ratio rather than the percentage of LDNs that can be more applicable in lung adenocarcinoma reflecting the immunosurveillance. This is not similar to another kind immunosuppressive cell MDSCs, which was deemed to share lots of characteristics to LDNs and extensively researched in lung cancer [8, 11, 21]. Our study provides an indicator showing the difference of LDNs and MDSCs

To test the distinction of phenotypes between HDNs and LDNs, expression levels of surface CD molecules of these neutrophils were compared. Similar to previous studies conducted in other diseases [10, 12, 15], the two groups of neutrophils from patients and healthy individuals express same membrane markers, CD15, CD45, CD11b and CD66b, and the expression levels are increased in LDNs. Although CD45 is not significantly higher in LDNs from healthy subjects, it is just an assistant marker to gate neutrophils in flow cytometry, so this marker is not in the range of discussion. Other three elevated expression markers suggest that LDNs are impaired activated neutrophils which have degranulated. CD11b and CD66b are presented on different granules [10, 22], and CD15 is also connected with degranulation [23], so they are associated with neutrophils activation. Meanwhile, the different phenotypes provide an evidence of diverse functions, such as arginase and ROS content [9, 11, 14].

Appropriate anti-cancer therapies are potential to inhibit immunosuppressive circuitries and reestablish immunosurveillance [24]. So whether LDNs in lung adenocarcinoma patients can be affected by treatment was explored. Indeed, the ratio of LDNs/HDNs from patients in the treatment period is close to healthy individuals while significantly lower than untreated new and recurrent cases, and the latter two groups of subjects are not obviously different. It is reasonable that newly diagnosed advanced patients harbored ill immune status reflected by higher LDNs/HDNs ratio, because immunosuppressive microenvironment is established concomitantly with neoplasm development [25]. For relapsing patients, the rebounded ratio could be regarded as a valuable mirror of disease progression. In contrast, lowered LDNs/HDNs ratio in patients undergoing treatment was likely benefited from anti-cancer therapies as mentioned above. The results may also guide future anti-tumor mode and provide a target for immunotherapy to reverse immunosuppression. Compared with traditional method, the use of targeted drugs Tyrosine kinase inhibitors (TKIs) in EGFR mutation-positive patients has achieved high clinical response rate [26, 27]. Besides, combined therapies improved outcomes and overcame drug-resistance [28, 29]. The evidence has been discovered in lung adenocarcinoma that changes in immune cells are related with gene mutation, implying the possibility of combination of TKIs and immunotherapies [17]. In this study, the ratio of LDNs/HDNs is significant higher in EGFR mutation-positive patients than negative ones, which is the basis for combined therapies.

Tumor immune is a complex network where numerous cells communicate each other frequently [30]. Prior literature also suggested a correlationship between neutrophils and lymphocytes [31]. Although CD8^+^ T cells are often referred as cytotoxic lymphocytes with tumor killer properties, current results showed the frequency of CD8^+^T cells is positively correlated with both frequency of LDNs in PBMCs and LDNs/HDNs ratio. A recent research indicated CTLs recruit MDSCs by collaborating with apoptosis-resistant tumor cells via Fas signaling [32]. This theory can serve as an explanation for present surprising phenomenon because certain similarities exist between LDNs and MDSCs. On the other hand, a potential immune escape mechanism of advanced lung cancer may be taken a glimpse here.

To further explore the upstream factors contributing to abnormal level of LDNs, two cytokines IL-17 and TGF-β from plasma were detected on the basis of early evidence [8, 18]. Unfortunately, although TGF-β is significantly higher in lung cancer patients, neither IL-17 nor TGF-β is associated with the dynamic change of frequency of LDNs or LDNs/HDNs ratio. A possible reason may be inappropriate storage of plasma sample, because the concentration of TGF-β detected in this research is obviously decreased as compared to previous studies [33, 34]. The uncorrelation between LDNs and the cytokines is also probably due to most of published experiments were carried out on animal model and breast cancer [8, 18]. So more work focusing on lung cancer is needed to be done in the future.

In conclusion, it is the first time to detect two phenotype distinct neutrophils, impaired activated LDNs and normal HDNs, in lung adenocarcinoma patients. We demonstrated significantly higher ratios of LDNs/HDNs appear in lung adenocarcinoma patients. And it revealed that the low ratio is associated with treatment history, negative EGFR mutation and lower level of CD8^+^ T cells. Nonetheless, the clinical significance of LDNs in disease stage, monitor and prognosis are required in future researches. The pathway resulting in LDNs generation is also necessary to clarify to develop corresponding intervention measures.

## MATERIALS AND METHODS

### Subjects and samples

Individuals with definite diagnosis of lung adenocarcinoma were recruited from Cancer Center, Union Hospital, Tongji Medical College, Huazhong University of Science and Technology (Wuhan, China). And only patients with loco-regional advanced or metastatic tumor were eligible. Exclusive factors included a history or concurrent status of autoimmune disease, asthma or AIDS. Healthy people without above diseases were recruited as control group. The study complied with the declaration of Helsinki and was approved by the Institutional Review Board of Tongji Medical College of Huazhong University of Science and Technology. All subjects gave written informed consent.

Peripheral blood was draw by venipuncture and collected in EDTA tubes. Samples from patients who were accepting anti-cancer therapy were obtained in treatment interval, more accurately, the day before conducting next cycle of therapy.

Peripheral blood mononuclear cells (PBMCs) and normal neutrophils fractions were isolated as described previously [35]. Briefly, each blood sample was diluted 1:1 with PBS (HyClone, Logan, Utah, USA). Then the mixture was slowly added to the top of equivoluminal Ficoll-Paque (Sigma, St. Louis, MO, USA). After centrifugation at 400 ´ g for 20 min at room temperature without brake, the PBMCs (including LDNs) were carefully collected from the interface between plasma and Ficoll. Normal neutrophils fractions (including HDNs) were separated from the granulocyte-erythrocyte sediment by hypotonic lysis (Becton-Dickinson, Franklin Lakes, NJ, USA). Both the two cell fractions were washed twice in PBS and resuspended in RPMI-1640 (HyClone, Logan, Utah, USA) for the further analysis.

### Flow cytometry

The following antibodies from Becton-Dickinson were used: FITC-conjugated anti-CD66b, PerCP-conjugated anti-CD45, APC-conjugated anti-CD15 and PE-conjugated anti-CD11b. According to the protocols, PBMCs or normal neutrophils fractions were incubated with these antibodies for 20 min at 4°C, then washed and resuspended in PBS. Isotype IgGs were used as controls. Analysis was performed on an FCM Calibur™ (Becton-Dickinson) and the results were analyzed using FlowJo 7.6.5 software (TreeStar, Inc, Ashland, Oregon).

To further explore the relationship between the two group cells and immune status, we detected the lymphocyte subgroups in peripheral blood of part patients with their consent, using these antibodies (Becton-Dickinson): FITC-conjugated anti-CD3, APC- conjugated anti-CD4, PE-conjugated anti-CD8, APC-conjugated anti-CD19, PerCP- conjugated anti-CD45, PE- conjugated anti-CD16/CD56.

### ELISA

Cytokine levels of human IL-17 and TGF-β in subjects’ plasma were determined using two ELISA kits (Dakewe, Shenzhen, China) following the manufacturer’s instructions. Samples were analyzed in duplicate, and the absorbance was compared with the standard curve to calculate the concentrations.

### Statistical analysis

Statistical analysis was performed with GraphPad Prism 5.0a (GraphPad, San Diego, California). Unless otherwise specified, the data are expressed as mean±SEM. Mann–Whitney test, Kruskal-Wallis test, Fisher’s exact test and Spearman’s rank test were used to determined statistical differences between variables when appropriate. A two-sided *P* < 0.05 was considered a significant difference.

## SUPPLEMENTARY MATERIALS FIGURE


