# Supplementary material for: Phenotypic and clinical characterization of low density neutrophils in patients with advanced lung adenocarcinoma
Source: Oncotarget. 2017 Jun 28;8(53):90969–78. doi: 10.18632/oncotarget.18771 (PMC5710898; doi:10.18632/oncotarget.18771)
Supplement: Supplementary file 1 [file oncotarget-08-90969-s001.pdf]

Phenotypic and clinical characterization of low density neutrophils in patients with advanced lung adenocarcinoma

Supplementary Materials

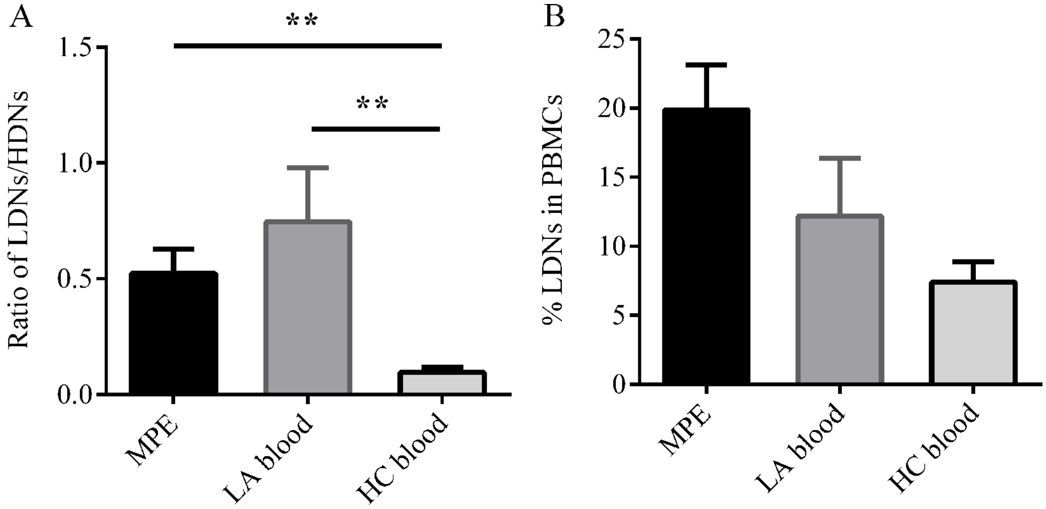

Supplementary Figure 1: Ratio of LDNs/HDNs and percentage of LDNs in PBMCs analyzed in malignant pleural effusion (MPE *n* = 4) and blood of lung adenocarcinoma (LA) patients, and blood of healthy controls (HC). LA patients include untreated and recurrent groups. Statistical significance was determined by a two-tailed Kruskal-Wallis test. $^{**}P < 0.01$.
